# Cyclosporin A induced toxicity in mouse liver slices is only slightly aggravated by Fxr-deficiency and co-occurs with upregulation of pro-inflammatory genes and downregulation of genes involved in mitochondrial functions

**DOI:** 10.1186/s12864-015-2054-7

**Published:** 2015-10-20

**Authors:** Ewa Szalowska, Tessa E. Pronk, Ad ACM Peijnenburg

**Affiliations:** RIKILT - Institute of Food Safety/Wageningen UR, Akkermaalsbos 2, P.O. Box 230, 6700 AE Wageningen, The Netherlands; Centre for Health Protection, National Institute for Public Health and the Environment (GZB, RIVM), Antonie van Leeuwenhoeklaan 9, 3721 MA Bilthoven, The Netherlands; RIKILT-Institute of Food Safety/Wageningen UR, Akkermaalsbos 2, 6708 WB Wageningen, The Netherlands

**Keywords:** Farnesoid X receptor (FXR), Precision cut liver slices (PCLS), Cyclosporine A, Hepatotoxicity, Mitochondrial functions, Inflammation, Peroxisome proliferator-activated receptor δ (PPAR δ), Transcriptomics

## Abstract

**Background:**

The transcription factor farnesoid X receptor (FXR) governs bile acid and energy homeostasis, is involved in inflammation, and has protective functions in the liver. In the present study we investigated the effect of Fxr deficiency in mouse precision cut liver slices (PCLS) exposed to a model hepatotoxicant cyclosporin A (CsA). It was anticipated that Fxr deficiency could aggravate toxicity of CsA in PCLS and pinpoint to novel genes/processes regulated by FXR.

**Methods:**

To test this hypothesis, PCLS obtained from livers of wild type mice (WT-PCLS) and Fxr-knockout mice (FXRKO-PCLS) were treated with 40 μM CsA for 24 h and 48 h. ATP and histological assays were applied to assess the viability of PCLS. DNA microarrays combined with bioinformatics analysis were used to identify genes and processes that were affected by CsA in WT-PCLS and/or FXRKO-PCLS. In addition, WT-PCLS and FXRKO-PCLS were exposed to the endogenous FXR ligand chenodeoxycholic acid (CDCA) and subjected to q-PCR to determine whether subsets of known FXR-targets and the identified genes were regulated upon FXR activation in an FXR-dependent manner.

**Results:**

No difference in viability was observed between WT-PCLS and FXRKO-PCLS upon CsA treatment. Transcriptomics data analysis revealed that CsA significantly upregulated stress-response and inflammation and significantly downregulated processes involved in lipid and glucose metabolism in WT-PCLS and FXRKO-PCLS. However, only in FXRKO-PCLS, CsA upregulated additional pro-inflammatory genes and downregulated genes related to mitochondrial functions. Furthermore, only in WT-PCLS, CDCA upregulated a subset of known FXR-target genes as well as the regulator of inflammation and mitochondrial functions peroxisome proliferator- activated receptor delta (Ppar delta).

**Conclusions:**

Although FXR governs energy metabolism, no major differences in response to CsA could be observed between WT-PCLS and FXRKO-PCLS in regulation of processes involved in lipid and glucose metabolism. This finding indicates that CsA does not directly affect FXR functions in relation to the above mentioned processes. However, the more pronounced induction of pro-inflammatory genes and the downregulation of genes involved in mitochondrial functions only in FXRKO-PCLS suggest that FXR deficiency aggravates CsA-induced inflammation and impairs mitochondrial functions. Therefore, FXR can exert its hepatoprotective functions by controlling inflammation and mitochondrial functions, possibly involving an FXR-PPAR delta cross-talk.

**Electronic supplementary material:**

The online version of this article (doi:10.1186/s12864-015-2054-7) contains supplementary material, which is available to authorized users.

## Background

Farnesoid X receptor (Fxr) is highly expressed in liver, intestine, kidney, adrenal glands and has a lower expression in white adipose tissue, pancreas, heart, and stomach [1]. Upon the discovery that bile acids (BA) are endogenous FXR agonists with chonedoexy cholic acid (CDCA) being the most potent FXR agonist, the primary functions of FXR were attributed to the maintenance of BA homeostasis [2]. FXR, upon activation by binding of a ligand, dimerizes either as a homodimer or as an heterodimer with another member of the nuclear receptor superfamily, retinoid X receptor (RXR), and binds to FXR response elements (FXRE) in the promoter region of its target genes to drive transcription [2]. FXR positively regulates the expression of several genes coding transporters and enzymes involved in BA homeostasis including bile salt export pump (BSEP), bile acid-CoA:amino acid N-acyltransferase (BAAT), or multidrug resistance protein 3 (MDR3). In addition, FXR can inhibit the expression of some of its target genes, including cholesterol-7α-hydroxylase (Cyp7a1), by a mechanism referred to as trans-repression involving induction of other transcription factors, such as small heterodimer partner (Shp). Thus, in liver FXR upregulates its direct target gene Shp, which in turn inhibits expression of the liver receptor homolog (Lrh)-1, liver X receptor (Lxr), and hepatocyte nuclear factor 4 α (Hnf4α), all necessary for constitutive expression of Cyp7a1, coding a rate limiting enzyme in the synthesis of BA from cholesterol [3] .

In recent years it has become evident that FXR, next to its well established role in BA homeostasis, is also involved in the maintenance of lipid and glucose metabolism [2]. Consistent with these observations, Fxr-knockout (KO) mice are dyslipidemic, with elevated plasma triglycerides and cholesterol levels [4]. With regard to regulation of glucose homeostasis, it was shown that Fxr-KO mice display peripheral insulin resistance implying that FXR is involved in regulation of insulin sensitivity and glucose metabolism [3]. In line with these findings, FXR was shown to regulate expression of the gluconeogenic genes such as phosphoenolpyruvate carboxykinase (Pepck) and glucose 6 phosphatase (G6Pase) [5, 6]. In addition to its role in BA and energy homeostasis, FXR exerts hepatoprotective functions through its anti-inflammatory, anti-fibrotic, anti-apoptotic, pro-regenerative, and detoxifying features [7].

Due to the complexity of hepatic FXR signaling, involving entero-hepatic communication and interplay with extrahepatic tissues [2], none of the current *in vitro* models allow to study FXR functions in full. However, it is possible to study mechanisms related to hepatic FXR signaling in *in vitro* liver models. One such model is represented by precision cut liver slices (PCLS). The major advantage of PCLS, compared to mono-cultures of hepatocytes, is the presence of parenchymal as well as non-parenchymal liver cells, whose interactions are important in the context of FXR signaling [8–11].

The objective of the present study was to investigate the role of FXR in the liver under a hepatotoxic challenge. It was anticipated that Fxr deficiency could aggravate effects of the model hepatotoxic compound cyclosporine A (CsA). CsA is an immunosuppressive drug commonly applied after solid organ transplantation to prevent rejection [12]. The pharmacological properties of CsA are related to repression of the activity of the immune system by interfering with T cell functioning [13, 14]. Adverse effects caused by CsA include hepatotoxicity that can lead to the development of cholestasis [15], fatty liver [16], and cardiovascular complications due to hyperlipidemia [17]. The primary mechanism of action underlying the hepatotoxicity of CsA is prevention of the mitochondrial permeability transition pore from opening leading to oxidative stress and impairment of mitochondrial functions [18]. This is most likely followed by induction of NFκB signaling driving expression of pro-inflammatory cytokines (e.g. TNFα, Il1α, and Il1β) and endoplasmatic reticulum (ER) stress, causing a disturbed vesicles formation necessary for protein, lipid, and bile acid trafficking [11, 19]. In addition, it was reported that expression of Fxr and its target genes was de-regulated upon treatment with CsA in different human and rodent *in vitro* liver models as well as rodents *in vivo* [11, 20–22].

In order to study the effect of Fxr deficiency under hepatotoxic challenge, mouse PCLS obtained from livers of wild type (WT) and Fxr-KO mice (referred to as WT-PCLS and FXRKO-PCLS, respectively) were treated with 40 μM CsA. ATP and histological assays were applied to assess the viability of the PCLS after 24 h and 48 h. DNA microarrays combined with bioinformatics analysis were used to identify genes and processes (i.e. pathways and gene ontology (GO) terms) that were affected in WT-PCLS and/or FXRKO-PCLS upon 24 h treatment with CsA. The genes and processes commonly affected in WT-PCLS and FXRKO-PCLS were considered as CsA targets, whose regulation was not dependent on FXR. The genes and processes that were significantly affected in either WT-PCLS or FXRKO-PCLS after CsA treatment were considered to be regulated in an FXR-dependent manner. To verify whether regulation of some of the identified genes was FXR-dependent, WT-PCLS and FXRKO-PCLS were exposed for 24 h to the endogenous FXR ligand (CDCA) and gene expression was analysed by q-PCR.

## Methods

### Chemicals

Cyclosporin A (CsA), chonedoexy cholic acid (CDCA), and bovine serum albumin (BSA) were purchased from Sigma (Sigma, Zwijndrecht, the Netherlands). Williams E medium (WEM), Glutamax, penicillin/streptomycin (pen/strep), D-Glucose, phosphate buffered saline (PBS) were obtained from Invitrogen (Invitrogen, Bleiswijk, the Netherlands).

### Preparation and incubation of liver slices

12-weeks old male C57BL/6 mice and 12-weeks old FXRKO mice on a C57BL/6 mice background were bred at the animal facility of University Medical Centre Groningen (UMCG), the Netherlands. FXRKO mice were constructed and tested as described previously [23]. After arrival to the animal facility of Wageningen University, animals were kept for 2 weeks at a housing temperature of 22 °C and at a relative humidity of 30–70 %. The lighting cycle was 12-h light and 12-h dark. At the age of 24 weeks animals were sacrificed by an overdose of isoflurane. The treatment protocol was approved by the Ethical Committee for Animal Experiments of Wageningen University.

Immediately after the animals were killed, the liver was perfused with PBS and placed in ice-cold Krebs–Henseleit buffer (KHB) (pH 7.4, supplemented with 11 mM glucose). Liver tissue was transported to the laboratory within approximately 30 min and cylindrical liver cores were produced using a surgical biopsy punch with diameter of 5 mm (KAI, SynErgo Europe, Romania). Liver cores were placed in a Krumdieck tissue slicer (Alabama Research and Development, Munford, AL, USA) filled with ice-cold KHB aerated with carbogen and supplemented with 11 mM glucose. Slices with a diameter of 5 mm, a thickness of 0.2 mm and a weight of approximately 6 mg were prepared. Immediately after preparation, slices were transferred into culture plates filled with pre-warmed (37 °C) WEM supplemented with pen/strep. Three liver slices were pre-cultured in one well of the 6-well plate filled with 4 ml of WEM for one hour with continuous agitation (70 rpm). Incubations were performed in an oxygen controlled incubator (Galaxy 48 R, New Brunswick, Nijmegen, the Netherlands) at 80 % of oxygen, 5 % CO_2,_and the remaining gas volume was filled up to 95 % with N_2_. After one hour of pre-incubation, media were removed and replaced with fresh media containing test compounds or appropriate solvents. After incubations, samples were snap-frozen in liquid nitrogen and stored in −80 °C for further analysis. Samples dedicated to histology were stored in 4 % formaldehyde at room temperature.

### PCLS exposure

For all exposure experiments, PCLS were obtained from 5 WT and 5 FXRKO mice. Slices obtained from each biological replicate were cultured apart and each culture consisted of 3 co-incubated slices. The exposure experiments were performed at 2 days; at day1, PCLS obtained from livers of 3 WT mice and 2 FXRKO mice were used and at day 2, PCLS obtained from livers of 2 WT mice and 3 FXRKO mice were used.

For the ATP assay and transcriptome analysis, WT-PCLS and FXRKO-PCLS were exposed to 40 μM CsA for 24 h. For histological examination, WT-PCLS and FXRKO-PCLS were exposed to 40 μM CsA for 24 h and 48 h. CsA was added to the culture medium as a stock solution in DMSO (final concentration of DMSO in the medium was 0.1 % v/v). Slices incubated with 0.1 % v/v DMSO served as control. These conditions were also used in a previous work studying the effects of CsA in mouse PCLS [11].

For q-PCR analysis, WT-PCLS and FXRKO-PCLS were exposed for 24 h to 100 μM of CDCA dissolved in the culture medium. This CDCA concentration was the same as used in a previous study investigating effects of CDCA in human PCLS [24]. PCLS incubated only in the culture medium served as control for CDCA exposure experiment.

### ATP assay

To assess PCLS viability after the exposure experiments, the ATP assay was performed. For each ATP assay three co-cultured slices were placed in 400 μL Cell Lytic MT buffer (Sigma, Zwijndrecht, the Netherlands). Slices were homogenized (6500 *g*, 8 °C) two times for 15 s using a tissue homogenizer (Precellys 24 Bertin Technologies, Labmakelaar Benelux B.V. Rotterdam, The Netherlands). To remove cellular debris, the homogenates were centrifuged for 5 min (14,000 g, 8 °C) and the remaining supernatant was divided into two portions of 200 μL. One portion was mixed with 100 μL of ATP lytic buffer from ATPlite kit (Perkin Elmer, Oosterhout, The Netherlands) for ATP measurements and the second portion was stored at −80 °C for protein measurements. ATP was measured as described by the manufacturer using a microplate reader (Synergy TM HT Multi Detection Microplate Reader, Biotek Instruments Inc, Abcoude, the Netherlands) with settings for luminescence 590/635 nm, top measurement, and sensitivity 230. ATP measurements were performed in technical duplicates and luminescence values were recalculated into μM ATP in total liver slices extracts.

The protein concentration was measured using the Bradford method (Protein assay, BioRad, Veenendaal, The Netherlands). Protein samples of 2 μL were diluted 80 times in PBS and measured according to the manufacturer’s protocol. BSA was used as a standard and each measurement was performed in duplicate. ATP concentration was normalized on mg of protein per slice. ATP concentration is the mean ± SD of 5 independent experiments.

### Histology

Besides measurement of ATP levels, also histological analysis was performed to examine the viability of the slices after incubation. Slices were exposed for 24 and 48 h to 40 μM CsA or DMSO. For the 48 h cultures, after 24 h the culture medium was replaced with fresh medium supplemented with CsA or DMSO. After incubation, slices were fixed in 4 % buffered formaldehyde and embedded in paraffin. Next, the paraffin cross-sections were prepared and stained with haematoxylin and eosin (HE) according to Mayer’s protocol [25]. Sections of 3 independent experiments per each group were analysed and photographed under a microscope with a 100-fold magnification.

### DNA microarray hybridizations

Gene expression analysis in PCLS incubated for 24 h with CsA or DMSO was performed using the HT Mouse Genome 430 PM array plate (s) using the Affymetrix GeneTitan system (Affymetrix, Santa Clara, CA, USA). RNA was extracted from co-cultured slices using the RNeasy Tissue Mini Kit (Qiagen, Venlo, The Netherlands) according to the manufacturer’s protocol. RNA concentration and purity were assessed using a NanoDrop ND-1000 spectrophotometer (Isogen IJsselstein, The Netherlands) by measuring absorption ratios at 260/280 and 230/280 nm. The integrity of the RNA samples was checked using the Shimadzu MultiNA Bioanalyzer (Shimadzu, Tokyo, Japan). Biotin- labelled cRNA was generated from total RNA with the Affymetrix 3’IVT Express Kit (Affymetrix, Santa Clara, CA, USA) according to the manufacturer’s instructions with an input of 100 ng total RNA. The Agilent Bioanalyzer (Agilent, Amstelveen, the Netherlands) and the Shimadzu MultiNA Bioanalyzer (Shimadzu,Tokyo, Japan) were used to examine the quality of cRNA to confirm that the average fragment size was according to Affymetrix’ specifications. For each sample, 7.5ug biotinylated cRNA was fragmented and hybridized in a final concentration of 0.0375 ug /ul on the Affymetrix HT Mouse genome 430 PM array (Affymetrix, Santa Clara, CA, USA). After washing and staining by the GeneTitan instrument (Affymetrix, Santa Clara, CA, USA) using the Affymetrix HWS kit for Gene Titan, absolute values of expression were calculated from the scanned array using the Affymetrix Command Console v 3.2 software. The data Quality Control was performed using the Affymetrix Expression Console v 1.1 (Affymetrix, Santa Clara, CA, USA) software to check whether all parameters met the quality specifications. The Probe Logarithmic Intensity Error Estimation (PLIER) algorithm method was used for probe summarisation [26].

In order to monitor the sample independent control and the performance of each individual sample during hybridization, hybridization controls were added to the hybridization mixture. To determine the biological variation between samples, the sample dependent controls such as internal control genes, background values, and average signals were used. In summary, all the data were within data Quality Control thresholds, according to the Affymetrix Expression Console specifications. Non-normalized data in a form of the Cell Intensity File (*.CEL) were re-annotated (EntrezGene htmg430pm_Mm_ENTREZG) and the data were RMA normalized [26]. The microarray data generated in this study were deposited to gene expression omnibus (GEO) and the GEO accession number is GSE63457 (data will be public upon acceptance of the manuscript).

### Identification of significantly affected genes

To identify genes significantly affected by CsA in WT-PCLS and FXRKO-PCLS, the normalized data were log2 transformed, followed by analysis of variance (ANOVA) with Benjamini-Hochberg correction for false discovery rate (FDR). Genes were considered significant if FDR < = 0.05 and fold change (FC) was ≤ or ≥ 1.5 for down-or up-regulated genes respectively.

### Data mining

#### Pathway analysis (MetaCore)

MetaCore identifies pathways using a default enrichment analysis. Genes significantly affected by CsA in WT-PCLS and FXRKO-PCLS (FDR ≤0.05) were uploaded to MetaCore for pathway analysis. The pathway analysis was performed using Functional Ontology Enrichment/Pathways Maps default option in MetaCore for mouse. The identified pathways were considered as significantly affected by the treatment after FDR correction if FDR ≤ 0.005.

#### Gene Ontology analysis (DAVID)

Genes significantly up- or down-regulated by CsA in WT-PCLS and FXRKO-PCLS were uploaded separately to Database for Annotation, Visualization, and Integrated Discovery (DAVID) Bioinformatics Resource. In DAVID, the Functional Annotation Clustering tool was used to identify over-represented up and down-regulated Gene Ontology (GO) terms [27, 28]. The Mouse Genome 430 2.0 was used as a background for the GO analysis. The GO terms after FDR correction (Benjamini-Hochberg) were selected at FDR ≤ 0.005 for further analysis and interpretation. In addition we selected genes significantly affected by CsA in WT-PCLS and FXRKO-PCLS identified in the significant GO terms for further analysis by hierarchical cluster analysis (HCA) and functional clustering (see below).

#### Hierarchical clustering analysis

The differentially expressed genes extracted from significant GO terms were analysed by hierarchical clustering analysis (HCA) using an open access bioinformatics tool Genesis (http://genome.tugraz.at/genesisserver/genesisserver_description.shtml). In the analysis gene expression values were log2 transformed and median centered, followed by HCA using average linkage clustering in Genesis.

#### Gene functional clustering (STRING)

Significant genes extracted from the significant GO terms were subjected to gene functional clustering with the open access bioinformatics tool STRING (Search Tool for the Retrieval of Interacting Genes/Proteins) version 8.2. STRING constructs functional networks using information from known and predicted protein-protein interactions present in curated as well as experimental databases [29].

### q-PCR

Expression of selected known and novel candidate FXR target genes was analysed in WT-PCLS and FXRKO-PCLS exposed to CDCA or control for 24 h by q-PCR. The q-PCR was performed using the Biorad CFX96 TM Real-Time Detection System (Bio-Rad, Veenendaal, The Netherlands) with the following cycling conditions: 15 min 95 °C followed by 40 cycles of 15 s 95 °C and 1 min 60 °C. Reactions were performed in 10 μl and contained 20 ng cDNA, 1× TaqMan PCR Master Mix (Applied Biosystems, Foster City, CA), and 250 nM probe and 900 nM primer (for actinβ) or 1×TaqMan gene expression assay (for the remaining genes) (Applied Biosystems, Foster City, CA). A specific primer set for actinβ (ACTB) was developed with Primer Express 1.5 (Applied Biosystems, Nieuwerkerk aan den IJssel, The Netherlands) and sequences were as follows, probe: TGT CCC TGT ATG CCT CTG GTC GTA CCA C, forward primer: AGC CAT GTA CGT AGC CAT CCA, and reverse primer: TCT CCG GAG TCC ATC ACA ATG. Primers for: small heterodimer protein (Shp), bile salt export pump (Bsep), fatty acid synthase (Fasn), cytochrome P450, Subfamily VIIA (Cyp7a1, acyl-Coenzyme A oxidase 2, branched chain (Acox2), acyl-Coenzyme A dehydrogenase, short/branched chain (Acadsb), ELOVL family member 6 (Elovl6), mitochondrial GTPase 1 homolog (Mtg1), aldehyde oxidase 1 (Aox1), peroxisome proliferator activator receptor delta (Pparδ), and peroxisome proliferator activated receptor gamma, coactivator 1 β (Ppargc1b) were purchased from Applied Biosystems. The commercial numbers of the Taqman assays and additional information about the tested genes is given in Additional file 1. Data were analyzed with SDS 2.0 software (Bio-Rad, Veenendaal, the Netherlands). For each sample, the RT-PCR reaction was performed in triplicate and the averages of the obtained threshold cycle values (C_T_) were processed for further calculations. To check for contaminating DNA, samples without reverse transcriptase (−RT reaction) were analysed as well. For normalization ACTB was used. Relative expression was calculated with ∆ (∆ (C_T_))-method [30]. Expression of each gene is the mean ± SD of 5 independent experiments.

### Statistical analysis

A Mann–Whitney (IBM SPSS Statistics version 20) was used to calculate differences between controls and slices cultured with CsA in normalized ATP content as well as to determine differences in gene expression measured by q-PCR between CDCA treated slices and controls. The cut off for statistical significance was set at a *p*-value ≤ 0.05.

## Results

### Assessment of slice viability

**Fig 1:**
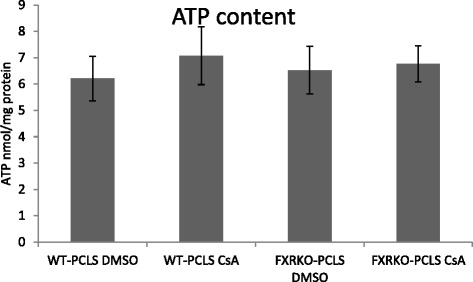
Viability of mouse liver slices upon treatment. Slices obtained from livers of WT and FXRKO mice (denoted as WT-PCLS and FXRKO-PCLS, respectively) were incubated for 24 h with 40 μM of cyclosporin A (CsA) or vehicle control (DMSO). ATP content (nmol/mg of protein) in slices treated with CsA was compared to control slices (DMSO). Each point is the mean ± SD of 5 independent experiments. None of the measured parameters was significantly affected

Viability of WT-PCLS and FXRKO-PCLS exposed to 40 μM of CsA or DMSO for 24 h was assessed by determining ATP levels normalized on total protein content. There was no statistically significant difference in the normalized ATP content between WT-PCLS and FXRKO-PCLS treated with DMSO or CsA indicating that exposure of both types of slices to 40 μM CsA did not result in cytotoxicity (Fig. 1).

**Fig. 2:**
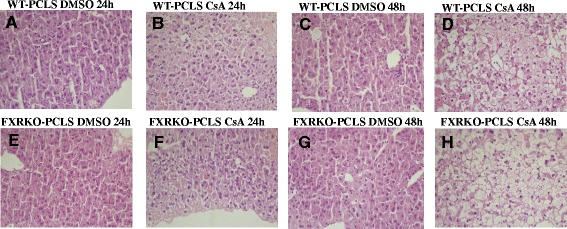
Histological analysis of liver slices treated with cyclosporin A (CsA). WT-PCLS (**a**-**d**) were cultured for 24 and 48 h in the presence of DMSO (**a** and **c**) or 40 μM CsA (**b** and **d**). FXRKO-PCLS (**e**-**h**) were cultured for 24 and 48 h in the presence of DMSO (**e** and **g**) or 40 μM CsA (**f** and **h**)

To determine whether CsA treatment differentially affected WT-PCLS and FXRKO-PCLS morphology, HE staining was performed. WT-PCLS and FXRKO-PCLS were exposed to DMSO or CsA for 24 or 48 h. There were no differences in morphology between WT-PCLS and FXRKO-PCLS cultured in the presence of DMSO for 24 and 48 h (Fig. 2a, e, c, and g respectively). There were also no differences between WT-PCLS and FXRKO-PCLS exposed to CsA for 24 and 48 h. However, the CsA treated slices developed ballooned hepatocytes in the outer layers of the slices (Fig. 2b and f, respectively). This phenotype was aggravated in both types of PCLS exposed to CsA for 48 h and there was no difference in the effect of CsA related to the number or time of appearance of ballooned hepatocytes between WT-PCLS and FXRKO-PCLS (Fig. 2d and h, respectively).

### Transcriptome analysis

**Fig. 3:**
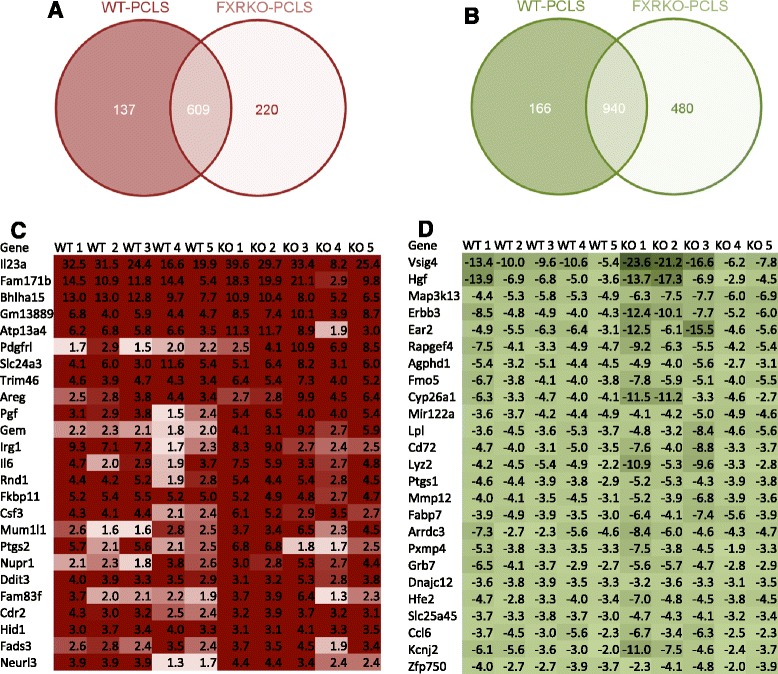
Identification of significantly affected genes. To identify genes significantly affected by CsA in WT-PCLS and FXRKO-PCLS, analysis of variance (ANOVA) was performed and corrected for FDR by the Benjamini test. Venn diagrams of significantly upregulated (FDR ≤0.05) and downregulated (FDR ≤ 0.05) genes in WT-PCLS and FXRKO-PCLS are presented in (**a** and **b**), respectively. The top 25 significantly upregulated and downregulated genes are presented as heat maps in (**c** and **d**), respectively. Each heat map shows fold change (treatment vs. control) calculated in individual experiments based on normalized gene expression values of DNA microarrays. WT-PCLS or WT indicates slices obtained from wild type (WT) mice. FXRKO-PCLS or KO indicates slices obtained form Fxr-knockout (FXRKO) mice. Red color indicates upregulation and green shows downregulation

Total RNA from WT-PCLS and FXRKO-PCLS treated for 24 h with 40 μM of CsA or DMSO were used for DNA microarray hybridizations and array data were analysed by ANOVA with FDR correction. The analysis resulted in the identification of 1549 genes that were significantly regulated (FDR ≤ 0.05, absolute FC ≥1.5) by CsA in WT-PCLS and FXRKO-PCLS in common (609 up- and 940 down-regulated) (Fig. 3a and b). It was observed that FCs in expression of these genes were comparable in both types of slices, indicating that quantitative effects of CsA were similar (GSE63457, Fig. 3c, and d). As shown in Fig. 3a and b, in addition to the genes commonly regulated by CsA in both WT-PCLS and FXRKO-PCLS, 303 genes were significantly affected only in WT-PCLS (137 up- and 166 down-regulated) and 700 genes were uniquely affected in FXRKO-PCLS (220 up- and 480 down-regulated).

### Functional analysis of genes commonly regulated by CsA in WT-PCLS and FXRKO-PCLS

**Fig. 4:**
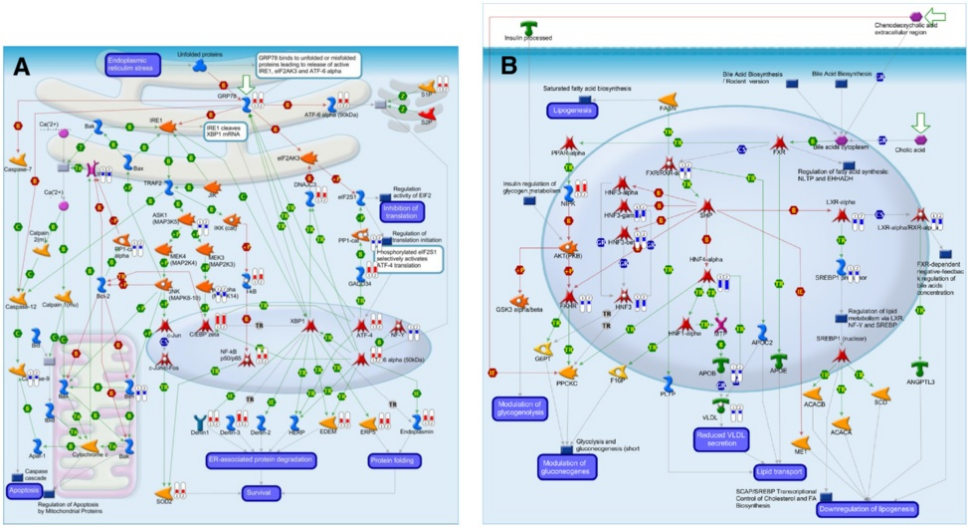
Effects of CsA on the expression of genes involved in endoplasmic reticulum stress and FXR-regulated pathways in WT-PCLS and FXRKO-PCLS. Pathway analysis was performed in MetaCore using as input significant genes (FDR ≤ 0.05) regulated by CsA in WT-PCLS and FXRKO-PCLS**.** Pathways were considered as significant if FDR ≤0.005. Among the most significantly regulated pathways were “Endoplasmic reticulum (ER) stress response pathway” (**a**) and “Bile acids regulation of glucose and lipid metabolism via FXR “(**b**). Blue and red bars indicate down-and up-regulation respectively of significantly affected genes. Numbers 1 and 2 below the bars indicate WT-PCLS and FXRKO-PCLS, respectively. Each bar represents average fold change of gene expression (treatment vs. control) in liver slices from five mice. For an explanation of the other MetaCore symbols is referred to http://pathwaymaps.com/pdf/MC_legend.pdf

The significantly affected genes were subjected to pathway analysis in MetaCore. The pathway analysis of the genes commonly regulated in WT-PCLS and FXRKO-PCLS upon exposure to CsA led to identification of 29 significant pathways (18 up- and 11 down-regulated at an FDR ≤ 0.005) (Table 1). All the identified pathways were grouped into functional categories based on information provided by the MetaCore classification tool. In general, it was observed that the significantly upregulated pathways clustered into “Endoplasmic reticulum stress”, “Metabolic diseases”, and “Inflammation” and that the most significantly upregulated pathway was represented by “ER stress response pathway “(Fig. 4a, Table 1). The significantly downregulated pathways could be clustered into functional categories such as “Coagulation” and “Regulation of energy metabolism” (Table 1). Examples of these pathways were “Blood coagulation”, (Table 1) and “Bile acids regulation of glucose and lipid metabolism via FXR” (Fig. 4b, Table 1).

**Table 1:** Pathway analysis of the commonly affected genes by CsA in WT-PCLS and FXRKO-PCLS

	Functional category	Pathways	FDR	In Data	Total
Upregulation	ER stress	Endoplasmic reticulum stress response pathway	2.0E-08	16	55
	ER stress	Role of PKR in stress-induced apoptosis	3.2E-05	12	53
	ER stress	CFTR folding and maturation (normal and CF)	3.4E-05	8	21
	ER stress	APRIL and BAFF signaling	2.0E-03	8	39
	ER stress	ATM/ATR regulation of G1/S checkpoint	3.3E-03	7	32
	ER stress	Regulation of degradation of deltaF508-CFTR in CF	1.7E-05	11	39
	ER stress	wtCFTR traffic/ER-to-Golgi (normal)	5.0E-04	6	15
	ER stress	DeltaF508-CFTR traffic/ER-to-Golgi in CF	5.0E-04	6	15
	ER stress	RAB1A regulation pathway	1.8E-03	5	12
	ER stress	DeltaF508-CFTR traffic/Sorting endosome formation in CF	1.8E-03	7	28
	ER stress	Regulation of degradation of wtCFTR	1.9E-03	6	20
	ER stress	Proteolysis_Role of Parkin in the Ubiquitin-Proteasomal Pathway	4.3E-03	6	24
	ER stress	Clathrin-coated vesicle cycle	4.6E-03	10	71
	Metabolic diseases	Role of free fatty acids in obesity and type 2 diabetes	2.6E-04	10	45
	Metabolic diseases	Role of ER stress in obesity and type 2 diabetes	9.9E-04	10	54
	Inflammation	IL-17 signaling pathways	1.8E-03	10	60
	Inflammation	CD40 signaling	3.0E-03	10	65
	Inflammation	Role of PKR in stress-induced antiviral cell response	4.4E-03	9	57
Downregulation	Coagulation	Thromboxane A2 pathway signaling	4.7E-04	13	49
	Coagulation	Lectin induced complement pathway	4.7E-04	13	49
	Coagulation	HTR2A-induced activation of cPLA2	1.1E-03	11	43
	Coagulation	Blood coagulation	1.8E-03	10	39
	Coagulation	Classical complement pathway	3.4E-03	11	52
	Coagulation	MIF - the neuroendocrine-macrophage connector	4.2E-03	10	46
	Coagulation	Alternative complement pathway	4.6E-03	9	39
	Regulation of energy metabolism	CREB pathway	2.1E-03	11	49
	Regulation of energy metabolism	Regulation of lipid metabolism by niacin and isoprenaline	3.7E-03	10	45
	Regulation of energy metabolism	Bile acids regulation of glucose and lipid metabolism via FXR	3.7E-03	9	37
	Regulation of energy metabolism	PPAR pathway	3.7E-03	12	63

Metacore pathway analysis using significant genes (FDR ≤ 0.05) regulated by CsA in WT-PCLS and FXRKO-PCLS identified 18 significantly upregulated pathways and 11 significantly downregulated pathways (FDR ≤ 0.005). The identified pathways are grouped into functional categories according to MetaCore pathway classification tool and are presented in the first column. Next columns represent names of the identified pathways, FDR score, number of significantly regulated genes identified in our study (“In data”), and the total number of genes belonging to the identified pathways (“Total”)

Next, genes significantly affected by CsA in WT-PCLS and FXRKO-PCLS were subjected to GO enrichment analysis in DAVID. The analysis identified that GO processes such as “ER”, “ER stress”, and “protein/vesicular transport” were significantly upregulated (FDR < 0.005; Table 2). While “coagulation”, “lipid metabolism”, and “protein processing” were significantly downregulated (FDR < 0.005; Table 2). Overall, the results of the GO analysis were in agreement with those of the MetaCore pathway analysis and pointed to regulation of similar biological processes including upregulation of ER stress and inflammation, and downregulation of coagulation and energy metabolism.

**Table 2:** Gene ontology (GO) analysis of the commonly affected genes by CsA in WT-PCLS and FXRKO-PCLS

	General function	GO process	FDR
Upregulation	ER	GO:0005783 ~ endoplasmic reticulum	2.8E-25
	ER	GO:0044432 ~ endoplasmic reticulum part	4.4E-15
	ER	GO:0031974 ~ membrane-enclosed lumen	3.0E-11
	ER	GO:0070013 ~ intracellular organelle lumen	5.6E-10
	ER	GO:0005788 ~ endoplasmic reticulum lumen	4.4E-09
	ER	GO:0042175 ~ nuclear envelope-endoplasmic reticulum network	2.5E-08
	ER	GO:0005789 ~ endoplasmic reticulum membrane	2.9E-07
	ER	GO:0012505 ~ endomembrane system	6.6E-05
	ER stress	GO:0006986 ~ response to unfolded protein	2.2E-04
	ER stress	GO:0034976 ~ response to endoplasmic reticulum stress	2.2E-03
	Protein/vesicular transport	GO:0045184 ~ establishment of protein localization	8.1E-08
	Protein/vesicular transport	GO:0015031 ~ protein transport	9.7E-08
	Protein/vesicular transport	GO:0008104 ~ protein localization	5.0E-07
	Protein/vesicular transport	GO:0031982 ~ vesicle	1.3E-03
	Protein/vesicular transport	GO:0016192 ~ vesicle-mediated transport	1.7E-03
	Protein/vesicular transport	GO:0046907 ~ intracellular transport	2.1E-03
	Protein/vesicular transport	GO:0016023 ~ cytoplasmic membrane-bounded vesicle	2.1E-03
	Protein/vesicular transport	GO:0031988 ~ membrane-bounded vesicle	2.5E-03
	Protein/vesicular transport	GO:0031410 ~ cytoplasmic vesicle	4.0E-03
Downreg.	Coagulation	GO:0009611 ~ response to wounding	2.4E-06
	Coagulation	GO:0006956 ~ complement activation	4.1E-06
	Coagulation	GO:0006958 ~ complement activation, classical pathway	9.8E-04
	Lipid metabolism	GO:0006644 ~ phospholipid metabolic process	5.0E-04
	Lipid metabolism	GO:0016042 ~ lipid catabolic process	8.6E-04
	Lipid metabolism	GO:0006631 ~ fatty acid metabolic process	9.6E-04
	Lipid metabolism	GO:0006775 ~ fat-soluble vitamin metabolic process	2.5E-03
	Protein processing	GO:0051605 ~ protein maturation by peptide bond cleavage	1.8E-05
	Protein processing	GO:0051604 ~ protein maturation	1.5E-04

GO analysis of the significant genes (FDR ≤ 0.05) regulated by Cyclosporin A in WT-PCLS and FXRKO-PCLS. GO analysis was performed in DAVID and GO process was considered as significant if FDR ≤ 0.005. In addition, the individual GO processes were grouped according to their general function and these groups are depicted in column denoted as “General function”

### Functional analysis of genes uniquely regulated in either WT-PCLS or FXRKO-PCLS

In order to better understand the impact of Fxr deficiency on CsA treatment, the genes uniquely regulated in WT-PCLS or FXRKO-PCLS were subjected to pathway and GO analyses. The pathway analysis did not identify any significant pathways (FDR ≤ 0.005) in neither WT-PCLS nor FXRKO-PCLS. However, with regard to GO analysis, GO- processes related to “ECM” were significantly downregulated (FDR ≤0.005) in WT-PCLS (Table 3). In FXRKO-PCLS, GO- processes related to “inflammation” were significantly upregulated, while GO- processes related to “mitochondrion” were significantly downregulated (Table 3).

**Table 3:** GO analysis of genes uniquely regulated in WT-PCLS or FXRKO-PCLS

	General process	GO process (WT-PCLS)	FDR	Direction of change
WT-PCLS	ECM	GO:0031012 ~ extracellular matrix	9.4E-10	Downregulation
	ECM	GO:0005578 ~ proteinaceous extracellular matrix	2.0E-09	Downregulation
	ECM	GO:0044421 ~ extracellular region part	1.5E-06	Downregulation
	ECM	GO:0005576 ~ extracellular region	2.0E-04	Downregulation
FXRKO-PCLS	Inflammation	GO:0005125 ~ cytokine activity	6.1E-05	Upregulation
	Inflammation	GO:0005615 ~ extracellular space	4.6E-03	Upregulation
	Inflammation	GO:0006935 ~ chemotaxis	9.8E-03	Upregulation
	Inflammation	GO:0042330 ~ taxis	9.8E-03	Upregulation
	Mitochondrial functions	GO:0005739 ~ mitochondrion	2.0E-11	Downregulation
	Mitochondrial functions	GO:0048037 ~ cofactor binding	1.5E-03	Downregulation
	Mitochondrial functions	GO:0044429 ~ mitochondrial part	2.9E-03	Downregulation

GO analysis of the significant genes (FDR ≤ 0.05) uniquely regulated by CsA in WT-PCLS or FXRKO-PCLS. GO analysis was performed in DAVID and GO process was considered as significant if FDR ≤ 0.005. In addition, the GO processes were grouped according to their general function and these groups are depicted in column denoted as “General function”. The last column informs whether a GO process was up- or down-regulated (“Direction of change”)

**Fig 5:**
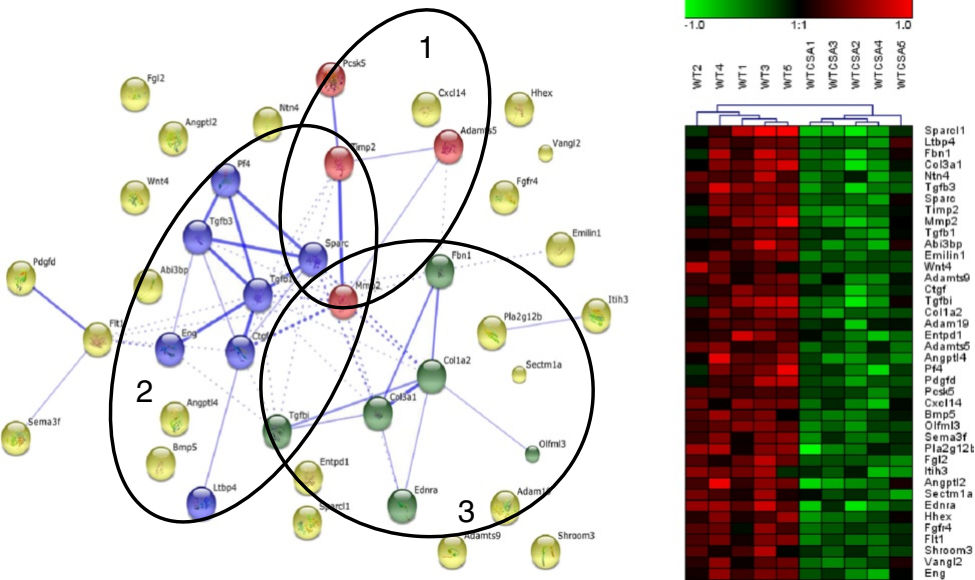
Functional and hierarchical clustering analysis of genes identified in GO processes related to “ECM” uniquely regulated in WT-PCLS. Genes identified in the significant GO-processes (FDR ≤ 0.005) related to “ECM” in WT-PCLS are presented as a network and a heat map. The network consists of 3 functional clusters shown as circles and denoted as: metalloproteinases (1), collagens (2), and development/morphogenesis (3). Connected nodes within the functional clusters indicate known gene-gene interactions and genes for which gene-gene interactions are unknown are presented as unconnected nodes. Thicker lines represent stronger associations between genes. Inter-cluster edges are represented by dashed lines. The heat map is the result of hierarchical clustering of log2, median centered, gene expression values in slices obtained from individual mice. Red and green indicate expression higher and lower, respectively, than the average expression of all samples within the heat map. WT stands for control (DMSO-treated) slices obtained from WT mice. WTCSA stands for CsA treated slices obtained from WT mice. KO stands for control (DMSO) slices obtained from FXRKO mice. KOCSA stands for CsA treated slices obtained from FXRKO mice

**Fig. 6:**
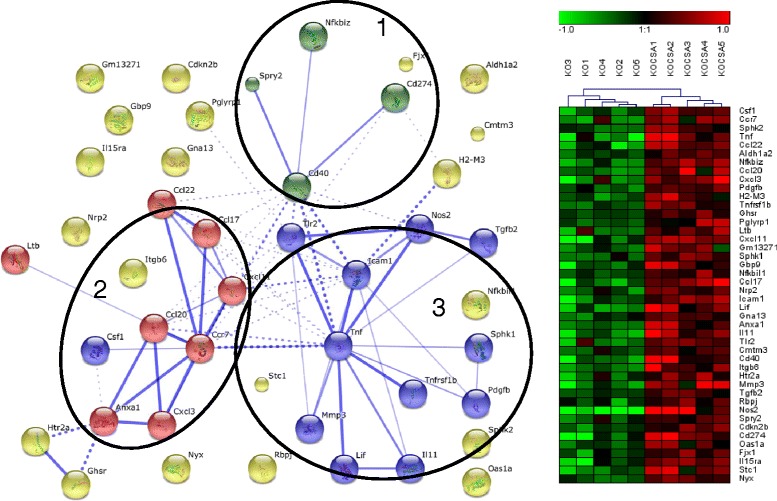
Functional and hierarchical clustering analysis of genes identified in GO processes related to “inflammation” uniquely regulated in FXRKO-PCLS. Genes identified in the significant GO-processes (FDR ≤ 0.005) related to “inflammation” in FXRKO-PCLS are presented as a network and a heat map. The network consists of 3 functional clusters shown as circles and denoted as: immunomodulation (1), chemokine signaling (2), and TNF signaling (3). The heat map is the result of hierarchical clustering of log2, median centered, gene expression values in slices obtained from individual mice. For explanation of the lines, spheres, the heat map, and abbreviations, see Fig. 5

**Fig. 7:**
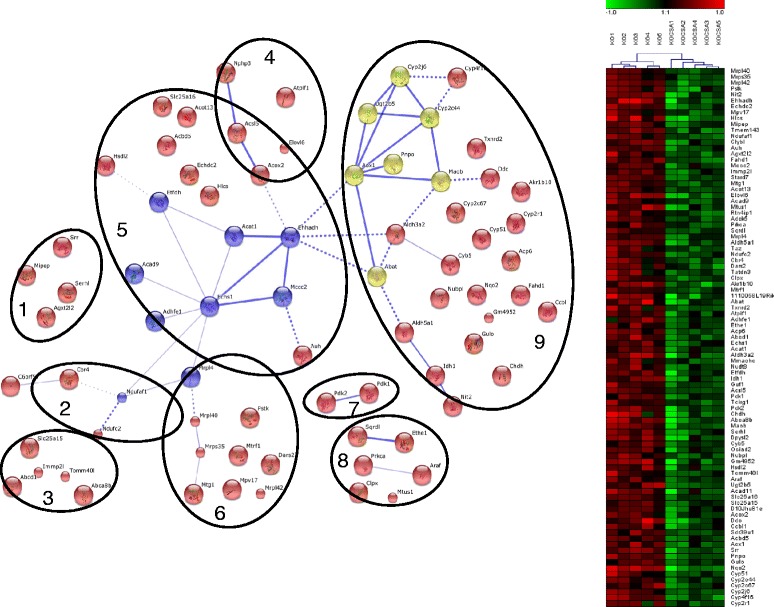
Functional and hierarchical clustering analysis of genes identified in GO processes related to “mitochondrion” uniquely regulated in FXRKO-PCLS. Genes identified in the significant GO-processes (FDR ≤ 0.005) related to “mitochondrion” in FXRKO-PCLS are presented as a network and a heat map. The network consists of 9 functional clusters shown as circles and denoted as: protein/amino acid (aa) processing n (1), NADH dehydrogenases (2), mitochondrial transporters (3), β-oxidation (4), CoA metabolism (5), mitochondrial ribosomal proteins (6), glucose metabolism (7), mitochondrial homeostasis (8), and xenobiotics metabolism (9). The heat map is the result of hierarchical clustering of log2, median centered, gene expression values in slices obtained from individual mice. For explanation of the lines, spheres, the heat map, and abbreviations, see Fig. 5

In order to gain more insight into functions of genes identified in the GO processes uniquely regulated in WT-PCLS or FXRKO-PCLS, genes significantly contributing to the GO enrichment were visualized as functional networks and heat maps. In WT-PCLS gene clusters related to development/morphogenesis and ECM components were identified (Fig. 5). In FXRKO-PCLS the genes upregulated by CsA were clustered into different pro-inflammatory processes, such as TNF and chemokine signaling (Fig. 6), while the downregulated clusters contained several genes involved in mitochondrial processes such as β-oxidation, CoA metabolism, and genes of the cytochrome P450 superfamily (Fig. 7).

### Fxr deficiency and GO-terms related to mitochondrial functions

**Fig. 8:**
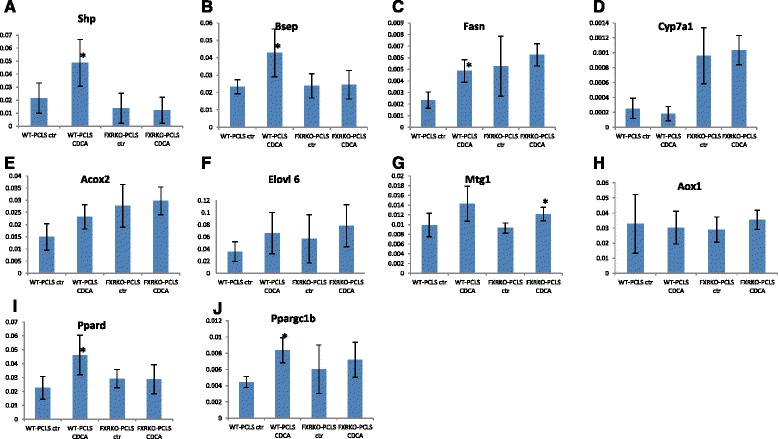
Validation of putative FXR target genes by q-PCR. WT-PCLS and FXRKO-PCLS were exposed for 24 h to chenodeoxycholic acid (CDCA) or vehicle control (ctr). Q-PCR was used to measure mRNA expression relative to actin β of known FXR target genes (Shp, Bsep, Fas, Cyp7a1, **a**-**d**) and putative FXR target genes (Acox2, Elovl6, Mtg1, Aox1, Pparδ, and Ppargc1b, **e**-**j**). Each condition (X-axis) is represented by 5 independent experiments. Relative gene expression compared to actin β is plotted on Y-axis. Asterisk indicates gene expression significantly different from controls according to Mann–Whitney-test (*p* < 0.05)

In a further analysis we focused on genes identified in the significant GO-processes related to mitochondrial functions, which were uniquely downregulated in FXRKO-PCLS upon CsA treatment (Table 3, Fig. 7). Based on these findings, we speculated that some of these genes might play a role in FXR signaling and/or represent novel FXR target genes. To determine whether a subset of these genes is regulated by endogenous FXR ligand in an FXR dependent manner, WT-PCLS and FXRKO-PCLS were exposed to CDCA and gene expression was analysed by q-PCR. As positive controls we measured the expression of known FXR target genes such as Shp, Bsep, Fasn, and Cyp7a1 [2]. As putative FXR target genes identified in the present study, we investigated the expression of Elovl6, Aox1, Mtg1, and Acox2. In addition, we tested Pparδ, since some of the genes downregulated by CsA in FXRKO-PCLS were previously described as Pparδ target genes i.e. Aox1and Ppargc1b [31]. As expected, in WT-PCLS, but not in FXRKO-PCLS, the known FXR target genes including Shp, Bsep, and Fasn were significantly upregulated by CDCA treatment 1.9, 2.1, and 2.1 fold, respectively (p < 0.05) (Fig. 8a-c). Surprisingly, despite significant upregulation of Shp there was no downregulation of Cyp7a1 in WT-PCLS treated with CDCA, Fig. 8d. Although the putative FXR targets i.e. Acox2 and Elovl6 tended to be upregulated by CDCA in an FXR-dependent manner, it did not reach a significant difference, Fig. 8e-f. Furthermore, it was observed that Pparδ and Ppargc1b were significantly induced by CDCA treatment in an FXR-dependent manner (2.3 and 1.9-fold respectively, p < 0.05), therefore suggesting that these genes are regulated by FXR, Fig. 8i-j.

## Discussion

In the present study we investigated the impact of Fxr deficiency on the effects of the model hepatotoxicant CsA in mouse PCLS. It was anticipated that Fxr deficiency could aggravate the hepatotoxicity of CsA and pinpoint to novel genes/processes regulated by FXR.

Measurement of cellular ATP levels revealed that the applied CsA concentration did not affect the viability of either WT-PCLS or FXRKO-PCLS, suggesting a comparable tolerance of both types of slices to 40 μM CsA (Fig. 1). Despite application of non-toxic concentrations of CsA, its treatment triggered ballooning of hepatocytes, which indicates on induction of inflammation and apoptosis in WT-PCLS and FXRKO-PCLS (Fig. 2). However, the ballooning occurred only in the outer layers of both types of PCLS which, compared to the inner layers, likely are exposed to higher CsA concentration. These findings are in agreement with previous work showing that the same phenotype developed upon treatment of mouse PCLS with CsA [11] and indicate that the response of WT-PCLS and FXRKO-PCLS to CsA is comparable.

The transcriptome analysis showed that in both WT-PCLS and FXRKO-PCLS, CsA upregulated pathways related to ER stress, protein/vesicular transport, NFκb signaling, and inflammation (Figs. 3c and 4a, Table 1). These findings are in line with the literature reporting that CsA induces oxidative stress, unfolded protein response, and inflammation [11, 19, 32]. However, the lack of major differences in response of WT-PCLS and FXRKO-PCLS to CsA seems to be in contrast to findings of Wang and colleagues who demonstrated that Fxr-deficiency in Fxr-KO mice and human hepatocytes have significantly increased hepatotoxicity upon exposure to other inducers of oxidative stress represented by carbon tetrachloride (CCl_4_) and hydrogen peroxide (H_2_O_2)_ respectively [33]. It was shown that oxidative stress induced by CCl_4_ or H_2_O_2_ inhibit the binding of FXR to FXRE by increased poly (ADP-ribosyl) ation of FXR catalysed by poly (ADP-ribose) polymerase 1 (PARP1), which alters FXR conformation leading to dissociation of FXR from FXRE and thereby suppresses expression of FXR target genes involved in hepatoprotection [33]. The lack of differences between FXRKO-PCLS and WT-PCLS with respect to regulation of stress response pathways and hepatotoxic effects in our study and the observations of Wang and colleagues could be explained by (i) differences in the mechanism of action between CsA and CCl_4_/H_2_O_2_ and/or (ii) model-dependent differences.

However, a recent study investigating effects of CsA in HepaRG cells showed that 50uM CsA induces oxidative stress as early as 15 min leading to irreversible alternations of efflux and uptake of BA co-occurring with disorganisation of F-actin microfilaments and bile canaliculi [34]. These early events were followed by altered expression of genes related to oxidative stress (at 6 h) and genes controlling bile acid homeostasis (at 24 h). Therefore, based on the results of Sharanek et al.[34], it could be anticipated that in our study the lack of differences in morphology between WT-PCLS and FXR-PCLS results from early and irreversible cytotoxic effects of CsA, which are gene-expression independent and cannot be compensated at later time points by potential hepatoprotective effects of FXR. From the other hand, the slightly worse hepatotoxic gene expression profile in FXRKO-PCLS vs. WT-PCLS, could reflect the disability of FXRKO-PCLS to involve the FXR-dependent hepatoprotective mechanism that controls expression of gens involved in inflammation and mitochondrial functions.

As mentioned above, we observed upregulation of several genes encoding pro-inflammatory cytokines such as Il-6, Csf3 (~3-6-fold upregulated), and Il-23. The latter gene was the most upregulated one in both types of PCLS (above 20 fold; Fig. 3c). IL-23 is produced by dendritic and Kupffer cells upon stimulation with pro-inflammatory stimuli e.g. LPS [35]. IL-23 together with IL-6 activates naive CD4 + T cells to differentiate into highly pro-inflammatory Th17 cells producing IL-17 that further enhances expression of IL-1, IL-6, TNFα, and NOS-2 [35]. However in our study, despite upregulation of Il-6 and Il-23, we did not observe upregulation of Il-17 in both kinds of slices upon CsA treatment. This finding indicates that CsA inhibited maturation of CD4 + T cells into Th17. However it is also possible that maturation of Th17 does not occur in PCLS, since CD4 + T cells might be absent in our model [36]. In this context it is noteworthy to mention that despite CsA therapy, there are patients with high serum levels of IL-23 and IL-17 suffering from acute organ rejection in contrast to patients with low serum levels of IL-23 and IL-17 who accept graft [37]. On the basis of these literature observations, it can be envisaged that in case the immunosuppressive effect of CsA (i.e. inhibition of CD4 + T/Th17 transition) is overruled by the hepatotoxic effects of CsA (i.e. stimulation of IL-23 expression), then CsA could induce the IL-23-dependent maturation of highly pro-inflammatory Th17 cells and trigger organ rejection.

Furthermore, WT-PCLS and FXRKO-PCLS significantly downregulated expression of key genes and pathways involved in regulation of energy metabolism such as Hnf4α, Lxrα, Rxrα, “Bile acids regulation of glucose and lipid metabolism via FXR”, and “PPAR pathway” (Fig. 4b, Table 1). These observations suggest that CsA can impair lipid and glucose homeostasis and are in line with literature reports linking CsA therapy with the development of metabolic diseases [15–17, 38]. These hepatotoxic effects can be related to CsA-driven upregulation of NFκb signaling and inflammation (Figs. 3c and 4a, Table 1), which are known to downregulate expression of different nuclear receptors and their target genes [24, 39, 40].

Moreover, in WT-PCLS and FXRKO-PCLS CsA significantly downregulated pathways related to coagulation (Table 1). This finding is in line with previously reported occurrence of haemorrhages in patients during CsA therapy [41].

Analysis of the genes uniquely downregulated by CsA in WT-PCLS revealed GO processes related to “ECM” (Table 3, Fig. 5). The fact that this downregulation occurred only in WT-PCLS points towards different effects of CsA on cellular signaling in the presence/absence of FXR. However, it is difficult to explain its exact meaning in the context of FXR signaling due to the lack of any literature data exploring effects of FXR on genes belonging to ECM.

Specifically in FXRKO-PCLS, CsA treatment upregulated GO processes related to “inflammation” and downregulated GO processes related to “mitochondrial functions” (Table 3, Figs. 6 and 7). With regard to the upregulation of GO processes related to “inflammation”, this finding is in line with known features of FXRKO mice, which display exaggerated response to pro-inflammatory stimuli [42, 43]. The significant downregulation of GO processes related to “mitochondrial functions” in FXRKO-PCLS points towards involvement of FXR in maintaining of mitochondrial functions. Consistent with this notion, recently it was reported that a synthetic FXR ligand 6-ethyl CDCA (6-ECDCA) upregulated several mitochondrial proteins in liver [44]. Moreover, Lee *et al*. reported that during oxidative stress, activation of FXR reduced mitochondrial dysfunction in hepatocytes [45]. Although, we did not confirm by q-PCR that the selected genes identified within the GO terms related to “mitochondrial functions” were significantly upregulated by CDCA in an FXR-dependent manner, we cannot exclude that such an upregulation could occur under certain physiological challenge (e.g. oxidative stress) or using an FXR-ligand other than CDCA. Based on our finding that the master regulator of mitochondrial functions and inflammation Pparδ and its target gene Ppargc1b [31, 46, 47] were significantly upregulated by CDCA in an FXR dependent manner (Fig. 8i-j), it can be speculated that the involvement of FXR in regulation of mitochondrial functions and inflammation could occur via an FXR-PPARδ crosstalk.

With regard to the application of mouse WT-/FXRKO-PCLS to study FXR signaling in the liver, this model seems to have some limitations. Although selected known FXR target genes, such as Shp, Bsep, and Fasn were significantly upregulated by CDCA in an FXR-dependent manner, we did not observe a downregulation of Cyp7a1 (despite significant upregulation of Shp). This finding indicates that PCLS might lack other factors involved in Cyp7a1 inhibition such as intestinal Fgf15 [48]. A similar phenomenon was observed in regulation of NTCP in human PCLS, where despite upregulation of SHP by CDCA, no repression of NTCP occurred [24]. These observations indicate therefore that PCLS may not be suitable to study genes regulated by trans-repression and/or cross-talk with extrahepatic tissues. However, our finding that Cyp7a1 basal expression was clearly higher in FXRKO-PCLS compared to WT-PCLS (Fig. 8d), indicates that features of FXRKO mouse are also preserved in PCLS [23].

## Conclusions

In summary, our study shows that, although FXR plays a central role in regulation of energy metabolism, no major differences in response to CsA could be observed between WT-PCLS and FXRKO-PCLS in regulation of processes involved in lipid and glucose metabolism. This finding indicates that CsA does not directly affect FXR functions in relation to the above mentioned processes. However, the CsA- induced upregulation of additional pro-inflammatory genes/processes and the downregulation of genes/processes involved in mitochondrial functions in FXRKO-PCLS only, indicate that FXR can be hepatoprotective by controlling inflammation and mitochondrial functions, possibly involving a crosstalk between FXR and PPARδ.

## Additional file

Additional file 1:
**Validation of known and putative Fxr target genes by q-PCR.** Information about primers and functions of genes used in q-PCR to test expression of known and novel putative FXR target genes in WT-PCLS and FXRKO-PCLS exposed to endogenous FXR ligand. Numbers of Taqman assays are given in column “Taqman assay”. Information about functions of the tested genes is according to Gene Cards http://www.genecards.org/. (PPTX 103 kb)

## Abbreviations

FXR: Farnesoid X receptor
PCLS: Precision cut liver slices
KO: Knockout
WT: Wild type
CsA: Cyclosporin A
BA: Bile acids
CDCA: Chenodeoxycholic acid
PPAR: Peroxisome proliferator- activated receptor
RXR: Retinoid X receptor
FXRE: Farnesoid X receptor response elements
BSEP: Bile salt export pump
BAAT: Bile acid-CoA:amino acid N-acyltransferase
MDR3: Multidrug resistance protein 3
Cyp7a1: Cholesterol-7α-hydroxylase
Shp: Small heterodimer partner
Lrh-1: Liver receptor homolog-1
Lxr liver: X receptor
Hnf4α: Hepatocyte nuclear factor 4 α
Pepck: Phosphoenolpyruvate carboxykinase
G6Pase: Glucose 6 phosphatase
ER: Endoplasmatic reticulum
(NF)κB: Nuclear factor κB
PBS: Phosphate buffered saline
DMSO: Dimethyl sulfoxide
KHB: Krebs-Henseleit Buffer
HEPES: (4-(2-hydroxyethyl)-1-piperazineethanesulfonic acid)
GO: Gene ontology
DAVID: The database for annotation, visualization, and integrated discovery
FDR: False discovery rate
SD: Standard deviation
RMA: Robust Multiarray Averaging
HCA: Hierarchical clustering analysis
PARP1: Poly (ADP-ribose) polymerase 1
ADP: Adenosine diphosphate
CCl_4_: Carbon tetrachloride
H_2_O_2_: Hydrogen peroxide
