# Supplementary material for: Cyclosporin A induced toxicity in mouse liver slices is only slightly aggravated by Fxr-deficiency and co-occurs with upregulation of pro-inflammatory genes and downregulation of genes involved in mitochondrial functions
Source: BMC Genomics. 2015 Oct 20;16:822. doi: 10.1186/s12864-015-2054-7 (PMC4617705; doi:10.1186/s12864-015-2054-7)
Supplement: Additional file 1: — Validation of known and putative Fxr target genes by q-PCR. Information about primers and functions of genes used in q-PCR to test expression of known and novel putative FXR target genes in WT-PCLS and FXRKO-PCLS exposed to endogenous FXR ligand. Numbers of Taqman assays are given in column “Taqman assay”. Information about functions of the tested genes is according to Gene Cards http://www.genecards.org/. (PPTX 103 kb) [file 12864_2015_2054_MOESM1_ESM.pptx]

## Slide 1
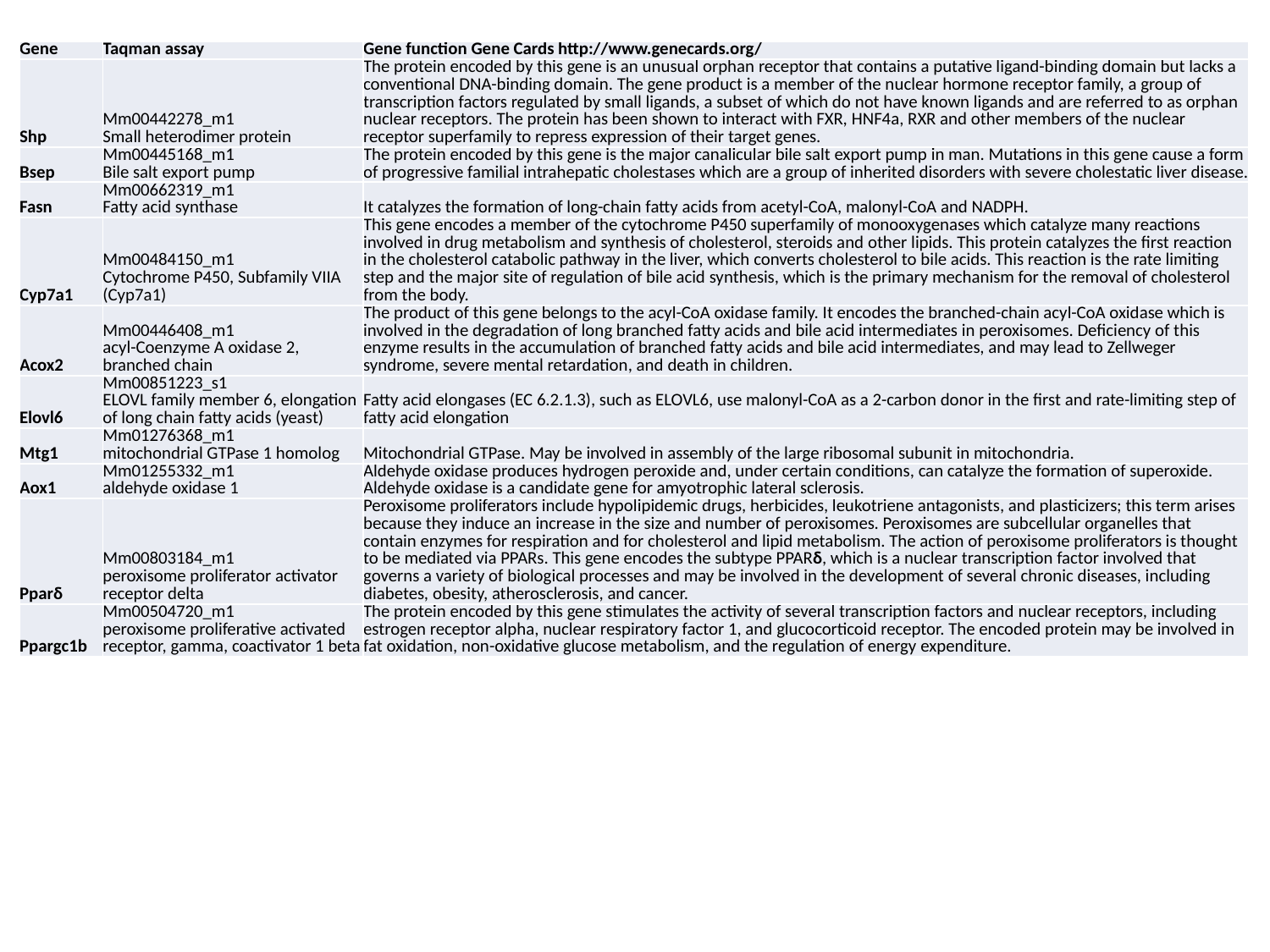

| Gene | Taqman assay | Gene function Gene Cards http://www.genecards.org/ |
| --- | --- | --- |
| Shp | Mm00442278\_m1 Small heterodimer protein | The protein encoded by this gene is an unusual orphan receptor that contains a putative ligand-binding domain but lacks a conventional DNA-binding domain. The gene product is a member of the nuclear hormone receptor family, a group of transcription factors regulated by small ligands, a subset of which do not have known ligands and are referred to as orphan nuclear receptors. The protein has been shown to interact with FXR, HNF4a, RXR and other members of the nuclear receptor superfamily to repress expression of their target genes. |
| Bsep | Mm00445168\_m1 Bile salt export pump | The protein encoded by this gene is the major canalicular bile salt export pump in man. Mutations in this gene cause a form of progressive familial intrahepatic cholestases which are a group of inherited disorders with severe cholestatic liver disease. |
| Fasn | Mm00662319\_m1 Fatty acid synthase | It catalyzes the formation of long-chain fatty acids from acetyl-CoA, malonyl-CoA and NADPH. |
| Cyp7a1 | Mm00484150\_m1 Cytochrome P450, Subfamily VIIA (Cyp7a1) | This gene encodes a member of the cytochrome P450 superfamily of monooxygenases which catalyze many reactions involved in drug metabolism and synthesis of cholesterol, steroids and other lipids. This protein catalyzes the first reaction in the cholesterol catabolic pathway in the liver, which converts cholesterol to bile acids. This reaction is the rate limiting step and the major site of regulation of bile acid synthesis, which is the primary mechanism for the removal of cholesterol from the body. |
| Acox2 | Mm00446408\_m1 acyl-Coenzyme A oxidase 2, branched chain | The product of this gene belongs to the acyl-CoA oxidase family. It encodes the branched-chain acyl-CoA oxidase which is involved in the degradation of long branched fatty acids and bile acid intermediates in peroxisomes. Deficiency of this enzyme results in the accumulation of branched fatty acids and bile acid intermediates, and may lead to Zellweger syndrome, severe mental retardation, and death in children. |
| Elovl6 | Mm00851223\_s1 ELOVL family member 6, elongation of long chain fatty acids (yeast) | Fatty acid elongases (EC 6.2.1.3), such as ELOVL6, use malonyl-CoA as a 2-carbon donor in the first and rate-limiting step of fatty acid elongation |
| Mtg1 | Mm01276368\_m1 mitochondrial GTPase 1 homolog | Mitochondrial GTPase. May be involved in assembly of the large ribosomal subunit in mitochondria. |
| Aox1 | Mm01255332\_m1 aldehyde oxidase 1 | Aldehyde oxidase produces hydrogen peroxide and, under certain conditions, can catalyze the formation of superoxide. Aldehyde oxidase is a candidate gene for amyotrophic lateral sclerosis. |
| Pparδ | Mm00803184\_m1 peroxisome proliferator activator receptor delta | Peroxisome proliferators include hypolipidemic drugs, herbicides, leukotriene antagonists, and plasticizers; this term arises because they induce an increase in the size and number of peroxisomes. Peroxisomes are subcellular organelles that contain enzymes for respiration and for cholesterol and lipid metabolism. The action of peroxisome proliferators is thought to be mediated via PPARs. This gene encodes the subtype PPARδ, which is a nuclear transcription factor involved that governs a variety of biological processes and may be involved in the development of several chronic diseases, including diabetes, obesity, atherosclerosis, and cancer. |
| Ppargc1b | Mm00504720\_m1 peroxisome proliferative activated receptor, gamma, coactivator 1 beta | The protein encoded by this gene stimulates the activity of several transcription factors and nuclear receptors, including estrogen receptor alpha, nuclear respiratory factor 1, and glucocorticoid receptor. The encoded protein may be involved in fat oxidation, non-oxidative glucose metabolism, and the regulation of energy expenditure. |
